# Supplementary material for: Incorporating uncertainty in learning to defer algorithms for safe computer-aided diagnosis
Source: Sci Rep. 2022 Feb 2;12:1762. doi: 10.1038/s41598-022-05725-7 (PMC8810991; doi:10.1038/s41598-022-05725-7)
Supplement: Supplementary file 1 — Supplementary Figure 1. [file 41598_2022_5725_MOESM1_ESM.docx]

**Supplementary File for Incorporating Uncertainty in Learning to Defer Algorithms for Safe Computer-Aided Diagnosis**

Jessie Liu^1^ Blanca Gallego^1^ Sebastiano Barbieri^1^

^1^Centre for Big Data Research in Health, University of New South Wales

**Results of Direct Triage by Ensemble Entropy**

**Supplemental Figure 1.** The results of the direct triage method when using a threshold value over the ensemble entropy, for the following tasks: (A) diagnosis of myocardial infarction, (B) diagnosis of any comorbidities, (C) diagnosis of pleural effusion and (D) diagnosis of pneumothorax. For each diagnostic task, the threshold value (x-axis) varies between the minimum and the maximum ensemble entropy measured across the entire patient group. Each panel shows the F1 scores (red line) for patients who are not deferred to human experts, ‘F1 Overall’ scores (red dashed line) and the corresponding defer rates (blue line). The diagnostic network F1 scores (without defer option) are also shown for each task (red dotted line).

Supplemental figure 1 shows that ensemble entropy alone cannot be used as the triage score to select patient groups associated with high F1 scores, because the entropy of the continuous probabilities computed by a deep ensemble [1] can be low for both correct and incorrect predictions. Across all tasks, the F1 scores decrease, rather than increase, as more patients are deferred for evaluation by a human expert. The system’s overall F1 score increases with the defer rate only because human experts are assumed to make correct diagnoses for all deferred patients.

**References**

[1] Lakshminarayanan, B., Pritzel, A., & Blundell, C. Simple and scalable predictive uncertainty estimation using deep ensembles. arXiv:1612.01474 [stat.ML] (2016).
